# Stomach intestinal pylorus sparing surgery (SIPS) with laparoscopic fundoplication (LF): a new approach to gastroesophageal reflux disease (GERD) in the setting of morbid obesity

**DOI:** 10.1186/s40064-015-1396-6

**Published:** 2015-10-13

**Authors:** Hinali Zaveri, Amit Surve, Daniel Cottam, Christina Richards, Walter Medlin, LeGrand Belnap, Samuel Cottam, Austin Cottam

**Affiliations:** Bariatric Medicine Institute, 1046 East 100 South, Salt Lake City, UT 84102 USA

**Keywords:** Duodenal switch, Fundoplication, GERD, Obesity, Weight loss, Reflux

## Abstract

The increase in the prevalence of obesity and gastroesophageal reflux disease (GERD) has paralleled one another. Laparoscopic fundoplication (LF) (Nissen or Toupet) is a minimally invasive form of anti-reflux surgery. The duodenal switch is a highly effective weight loss surgery with a proven record of long term weight loss success. However, fundoplication alone does not give satisfactory results when used for GERD in morbidly obese patients. Here we present a novel approach combining stomach intestinal pylorus sparing surgery (SIPS) with LF for morbidly obese patients with GERD. The data from patients who underwent the SIPS procedure along with LF in past year was retrospectively analyzed. The variables collected were age, sex, height, weight, intra-operative and post-operative complications, length of stay, operative time, and estimated blood loss. All revisions were excluded. Descriptive statistics such as mean and standard deviation were used to analyze the data. The total sample size of the study was 5 patients, with a mean age of 59.6 ± 16.4 years, a mean weight of 292.1 ± 73.6 lbs., and a mean body mass index (BMI) of 43.4 ± 6.3. Weight loss patterns were the same as those without LF. All the 5 patients had resolution or improvement in their GERD symptoms within 6 months. SIPS with LF provides substantial and sustained weight loss and GERD resolution. Long term follow ups and further study on this novel surgical technique is recommended.

## Background

Morbid obesity is a chronic disease which leads to progressive co-morbidities, socio-economic problems, undesirable quality of life, and earlier death. To date only metabolic/bariatric surgeries have achieved significant weight loss, with the corresponding correction or improvement of co-morbidities, improving quality of life. Duodenal switch (DS) is one of the most efficacious forms of bariatric surgical therapy for the morbidly obese available to the clinician (Hess et al. 2005; Buchwald et al. 2004).

Gastroesophageal reflux disease (GERD) is a common comorbidity associated with obesity (Hampel et al. 2005). However, laparoscopic fundoplication (LF) does not give satisfactory results in treatment for GERD in morbidly obese patients since obesity predisposes the patient to high rates of LF failure (Antanavicius et al. 2008; Makris et al. 2009).

We report a novel technique involving both LF and a modification of the DS called SIPS (stomach and intestinal pyloric sparing surgery) which together provide optimal weight loss and optimal anti-reflux characteristics while limiting complications associated with another common weight loss intervention for GERD in the setting of morbid obesity(the laparoscopic gastric bypass [LRYGBP]).

## Method

This is a retrospective analysis of the initial experience from a single surgeon at single institution. The primary objective of this study was to evaluate the SIPS procedure along with LF in terms of weight loss, operative complications, and GERD resolution.

Each patient had a thorough work up for morbid obesity and GERD. All the patients were experiencing GERD symptoms before surgery, and underwent esophagogastroduodenoscopy (EGD) and a transnasal endoscopy (TNE) to assess GERD symptoms and erosive esophagitis (EE). Two patients had a Bravo pH study and manometry to asses for esophageal dysmotility and to confirm the diagnosis of GERD. The other three patients had giant hiatal hernias on endoscopy with erosive esophagitis. Therefore, we did not do pH studies or manometry as those diagnostic tests don’t work well in the setting of giant hiatal hernias. Additionally, erosive esophagitis is a clear indicator of GERD invalidating the need for pH studies. All patients were required to provide written informed consent for both the fundoplication and the SIPS procedure before undergoing surgery. All the procedures were performed using a laparoscopic approach. Each patient had a post-operative upper gastro-intestinal (UGI) series prior to leaving the hospital to assess for leaks and obstructions and the adequacy of the wrap. All patients were seen back in clinic for follow up at 1 week, 1 month, 3 months, 6 months, and 1 year. The GERD-HRQL questionnaire was used to assess typical symptoms of GERD.

Descriptive statistics were used to calculate the mean and standard deviation of pre-operative characteristics such as age, weight, height, and BMI. Procedure time was gathered and started with the first incision and ended with the dressing.

Our research was carried out according to our institutions guidelines and the permission was granted to us to assess the patient’s data.

### Surgical technique

Each case begins with placement of four trocars and a liver retractor. Next the short gastric vessels are taken down. This facilitates the dissection of the paraesophageal hernia repair. The entire sac is removed using blunt and sharp dissection using an ultrasonixs dissector (Covidien LLC). This dissection is carried superiorly until approximately 5 cm of intra-abdominal length is achieved. The hernias are all repaired posterior to the esophagus with a two layer running technique with an endostich and 2.0 surgidac sutures. The first layer is a deep layer that starts where the crus meets inferiorly and goes up to the base of the esophagus. Once the esophagus is reached and we check to make sure there is no anterior defect, the suture line is run down back to the starting point and tied to the end of the stitch. This repair is reinforced with a PTFE felt mesh or Pariatex mesh (Covidien Corp.). Then a 40 French Bougie is placed. Depending on the patient, the wrap is created in a fashion described by Toupet or Nissen.

Next a Sleeve Gastrectomy is performed. The lesser sac is entered 4–6 cm from the pylorus. Then an Endo GIA (Covidien) stapler is fired along the previously placed sizing tube. Once the staple line reaches the prior LF, the staple line deviates laterally attempting to resect as much of the fundus as possible. Blood vessels to the lesser curve are persevered ensuring adequate blood supply.

The next step is to divide the duodenal bulb. This is done by taking down all the gastro-epiploic vessels from the end of the sleeve dissection to past the pylorus. A band passer is then placed towards the liver under the duodenum, and a window is made through the duodenal-hepatic ligament. Once the window is created, an Endo GIA (Covidien) stapler is passed around the duodenum and divides the duodenum 3 cm from the pylorus circumferentially. The distal duodenal stump is then sewn over with absorbable suture.

**Fig. 1:**
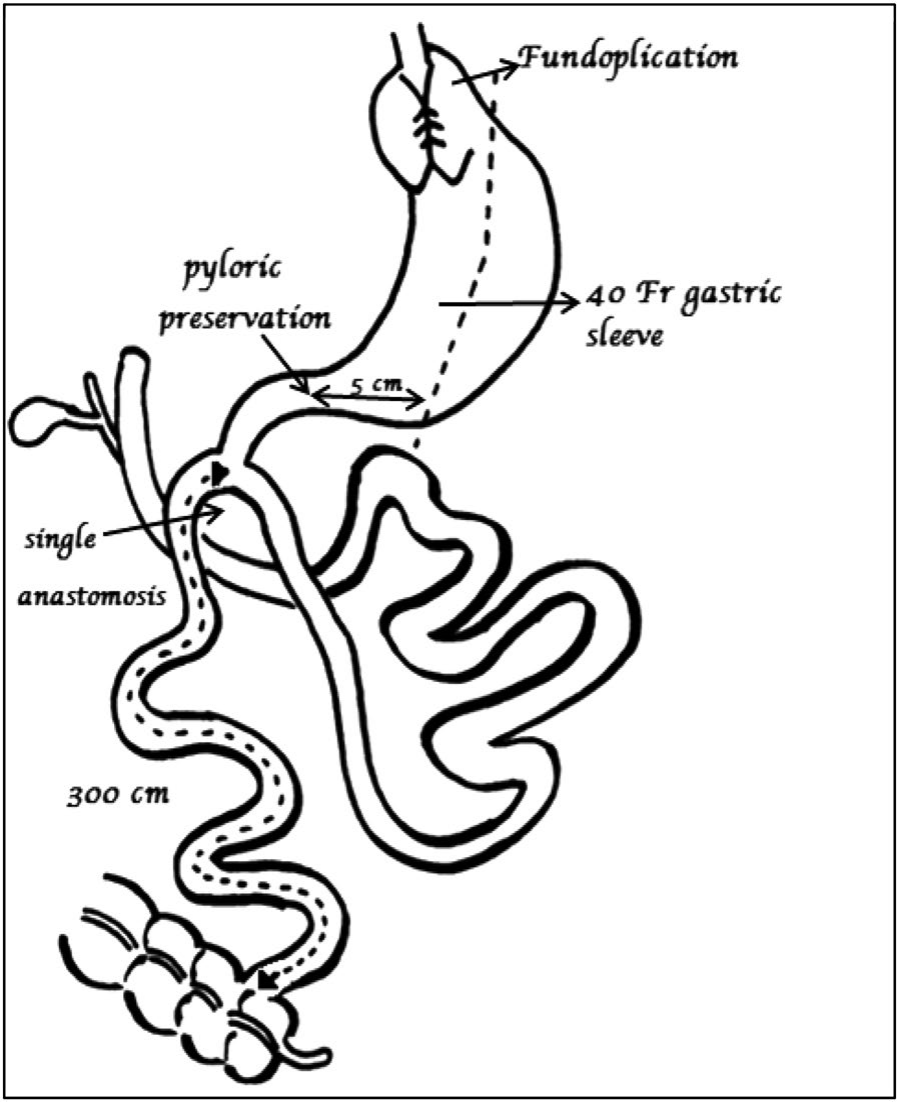
Diagrammatic representation of SIPS with Nissen Fundoplication

Next the ileo-cecal valve is located and traced retrograde to 300 cm, and that point is brought up and sewn to the proximal duodenal staple line. The loop limb is sewn to the proximal duodenal stump using 2.0 Polysorb (Covidien). Enterotomies are made in both the limbs and 3.0 Polysorb is used to do another posterior row and anterior row (Fig. 1).

The anastomoses are tested intra-operatively with pressurized air to check for leaks. The resected portion of the stomach is taken out of the abdominal cavity. Antibiotics and deep vein thrombosis (DVT) prophylaxis are used in all patients. The patients are discharged the next day if they do not exhibit nausea, vomiting or any other discomfort.

### Classification of erosive esophagitis

All patients receive preoperative endoscopic evaluation. The diagnosis and classification is based on Los Angeles (LA) classification (Lundell et al. 1999). Table 1 explains the classification.

**Table 1:** Los Angeles classification for erosive esophagitis

Grade	Definition
A	≥1 mucosal breaks ≤5 mm long, none of which extends between the tops of the mucosal folds
B	≥1 mucosal breaks >5 mm long, none of which extends between the tops of mucosal folds
C	Mucosal breaks that extend between the tops of ≥2 mucosal folds, but which involve <75 % of the esophageal circumference
D	Mucosal breaks which involve ≥75 % of the esophageal circumference

### Grading of the gastroesophageal valve

Endoscopic grading of the gastroesophageal valve provides useful information about the reflux status (Öberg et al. 1999). To evaluate the same, we use the Hill Valve Grading Scheme (Hill et al. 1996). The geometry of the gastroesophageal valve is assessed with the endoscope in the retroflexed position. The presence and appearance of the gastroesophageal flap valve is graded as I through IV.

Grade I gastroesophageal valve—defined by the presence of a prominent fold of tissue closely approximated to the shaft of the endoscope and extending 3–4 cm along the lesser curve at the entrance of the esophagus into the stomach.

Grade II gastroesophageal valve—The fold of tissue is less prominent, and there are occasional periods of opening and rapid closing around the endoscope with respiration.

Grade III gastroesophageal valve—There is no prominent fold at the entrance of the esophagus into the stomach, and the endoscope is not tightly gripped by the tissues.

Grade IV gastroesophageal valve—The patient has a large hiatal hernia and essentially no fold where the lumen of the esophagus is gaping open, allowing the squamous epithelium to be viewed from below.

### Measurement of reflux symptoms

The GERD-Health Related Quality of Life Questionnaire (GERD-HRQL) is a self-assessed, reliable, valid, responsive, and practical measure of symptom severity in patients with GERD (Velanovich et al. 1996; Velanovich 2007). It’s a 10-item questionnaire with an assessment of overall satisfaction. Items are scored on a 6 point scale (0–5), addressing domains like heartburn (Items 1–6), dysphagia (item 7), odynophagia (item 8), meteorism (item 9), effect of medications (item 10), and satisfaction level. But no extra-esophageal symptoms are included. It is evaluated post operatively at 6 months and 1 year. The heartburn score is calculated by summing the individual score to questions 1–6, with score of 30 being the worst heartburn and score of 0 being no heartburn. Any score of ≤12 with each individual question not exceeding 2 indicates heartburn elimination. The total score is calculated by summing the individual scores to questions 1–10, with greatest possible score of 50 (worst symptoms) and lowest possible score of 0 (no symptoms) (Hunter et al. 1996).

## Results

We analyzed a total of 5 patients aged between 18-85 years. The pre-operative characteristics are shown in Table 2. The mean operative time overall was 115 min. The mean estimated blood loss was 35 cc. The mean post-operative hospitalization stay was 2.4 ± 1.14 days. Two patients (40 %) had perioperative complications. One patient had an ileus, while the other had low oxygen saturation on day 2. However, both the complications were mild and patients recovered uneventfully. The first patient returned within 30 days of the surgery with nausea, vomiting, diarrhea, and clostridium difficile colitis. Only one (20 %) reported dysphagia (Post op >30 days). Table 3 highlights operative outcomes.

**Table 2:** Preoperative characteristics

No. of patients	5
Age (years)^a^	59.6 ± 16.4
Weight (lbs.)^a^	292.1 ± 73.6
Height (in)^a^	68.3 ± 4.2
BMI^a^	43.4 ± 6.3
Male	3
Female	2
Ideal Body Weight (lbs.)^a^	147.5 ± 26.1
Excess body Weight (lbs.)^a^	144.6 ± 51.5

^a^Values are expressed as mean ± SD

**Table 3:** Post-op complications

Ileus	1
Low oxygen saturation	1
Nausea with Diarrhea (Clostridium Difficile Colitis)	1
Dysphagia requiring intervention	1
Readmission <30 days	1

### Weight loss analysis

Each patient’s weight loss mirrored our larger group of SIPS patient. Refer to Table 4 for complete analysis.

**Table 4:** Weight loss analysis

Months	<3 months (n = 5)	3–6 months (n = 5)	7–12 months (n = 2)	>12 months (n = 2)
Total body weight loss (lbs.)ª	42.1 ± 11.6	69.9 ± 30.1	86.5 ± 14.1	98.9 ± 13.1
Total body weight loss (%)ª	14.5 ± 2.3	23.4 ± 5.2	33.1 ± 0.2	38 ± 1.5
Excess weight loss (%)ª	29.9 ± 5.8	47.9 ± 8	73.5 ± 5.3	84.2 ± 3.4
BMI reduction (kg/m2)ª	6.3 ± 1.2	10.3 ± 3.5	13.2 ± 0.1	15.1 ± 0.4
Excess BMI loss (%)ª	35.8 ± 9.1	56.8 ± 11.5	89.1 ± 2.7	102.2 ± 6.4

^a^Values are expressed as mean ± SD

### GERD analysis

Refer to Table 5 for complete pre-operative and post-operative GERD analysis.

**Table 5:** GERD analysis

Subjects	Pre-operative TNE + EGD			Surgery	Post-operative GERD-HRQL Scale	Extra notes
	LA Classification	Grading of GE Valve	Hernias			
1	D	IV	5 cm Sliding Hiatal Hernia	SIPS + HHR with Mesh + NF	0	Pre-operative Bravo PH study was positive for abnormal distal esophageal acid exposure DeMeester score-105.5
2	A	IV	8 cm Sliding Hiatal Hernia	SIPS + NF	19	Patient had history of esophageal dysmotility syndrome Post-operative manometry at 3 months showed poor esophageal dysmotility which was contributed to past history of motility disorder
3	C	IV	5 cm Sliding Hiatal Hernia	SIPS + HHR with Mesh + NF	1	Pre-operative bravo pH study was positive for abnormal distal esophageal acid exposure DeMeester score was 23.6
4	D	IV	6 cm Sliding +huge paraesophageal hernia	SIPS + Paraesophageal Hernia Repair with Mesh + complete fundoplication	9	
5	B	II	Huge paraesophageal Hernia	SIPS + Paraesophageal hernia repair with mesh + NF	2	Upper GI series at 6 months showed intake Nissan with no reflux

*HHR* hiatal hernia repair, *NF* Nissen fundoplication

Table 6 summarizes the post-operative GERD-HRQL scale.

**Table 6:** Post-operative GERD-HRQL SCALE

Patients no.	Heartburn Score (Q 1–6)	Dysphagia	Odynophagia	Meteorism	Effect of medications	Total score	Satisfaction level
1	0	0	0	0	0	0	Satisfied
2	10	2	2	5	0	19	Satisfied
3	0	0	0	1	0	1	Satisfied
4	6	0	0	3	0	9	Satisfied
5	0	0	0	2	0	2	Satisfied
Mean	3.2	0.4	0.4	2.2	0	6.2	Satisfied

## Discussion

The increasing prevalence of obesity worldwide has coincided with an increasing prevalence of GERD (Hampel et al. 2005). Morbid obesity is a key risk factor for GERD partly because obesity increases the intra-abdominal pressure, generating the forces necessary to cause reflux (Hampel et al. 2005; Barak et al. 2002; Groot et al. 2009). Obese patients may have an increased risk for hiatal hernia, which has a role in initiating and promoting GERD (Kahrilas 1999). Although LF is not the only treatment modality for intractable GERD, it is the preferred method for most surgeons.

Several studies have suggested that Roux-en-Y gastric bypass is a more effective operation for GERD than Nissen fundoplication in the morbidly obese GERD patients (Braghetto et al. 2012; Varela et al. 2009). While proponents are correct to argue that LRYGBP does eliminate GERD in most patients and it is a successful weight loss operation, the gastric bypass surgery yields significant long term complications associated with the creation of the Roux limb such as ulcers, strictures, intussusceptions, dysfunctional Roux limb syndrome, internal hernias, slippage of the gastric pouch, and dumping syndrome. The SIPS with LF is not subject to these side effects (Iannelli et al. 2006; Ledoux et al. 2014; Gasteyger et al. 2008; Zellmer et al. 2014). Because of these side effects, Gastric Bypass may not be an ideal operation for all morbidly obese GERD patients especially for patients who require long term NSAID therapy which is associated with bleeding ulcers postoperatively.

Laparoscopic Sleeve Gastrectomy (SG) is one of the most commonly used Bariatric Surgeries. However, SG alone may cause or worsen GERD, as mentioned in the literature (Santoro et al. 2014; Tai et al. 2013; Peterson et al. 2012). Obesity itself also worsens GERD symptoms and causes fundoplication to fail. To achieve adequate weight loss and adequate GERD control in this patient population, we proposed combining two procedures that already provide good disease treatment for both disease processes present.

SIPS is a new technique that is a slight modification of the SADI (Single Anastomosis Duodenal-Ileal bypass) (Sanchz-Pernaute et al. 2010). SIPS differs in that a smaller bougie is utilized, and intestinal length (3 m) is kept longer. The longer length along with the ileo-cecal valve helps reduce short bowel syndrome. The preservation of the pylorus provides control of solid emptying reducing the chances of dumping syndrome and assisting in maintaining a physiologic based rate of gastric emptying.

However the question that remains is whether this information is of importance to practicing bariatric surgeons and Gasto-surgeons for treating GERD in morbidly obese patients or whether another type of modification is necessary? This was answered recently by Khazzaka and Sarkis (2013) who reported fundoplication with gastric plication in 16 patients. Excess weight loss at 1 year was 10 kgs with resolution of GERD in all 16 patients. Thereafter, surgeons from Taiwan published a paper on Nissen fundoplication with Gastric Plication (greater curvature side plication) in 25 patients with the excess weight loss of 24.6 kgs at 12 months (Lee et al. 2014). However 8 % of their patients had a major complication post operatively. 21 patients out of 25 had improvement with their GERD symptoms. With our technique we observed that our excess weight loss between 3 and 6 months was 69.980 lbs (31.81 kgs) and between 6 and 12 months, it was 86.50 lbs (39.32 kgs) which is more than both the studies with gastric plication.

Indeed a patient losing weight helps improve GERD, but there are some other theoretical explanations for this improvement that can occur before weight loss (Peterson et al. 2012). By performing the SG as a first procedure of SIPS, we remove most oxyntic cells which reduces acid production. (Although it might be obvious, it has never been properly proved.) Even the faster rate of gastric emptying for liquids that is observed with sleeve helps in preventing and treating GERD. Additionally, we have data about the effect of LF on transient LES relaxations (TLESRs). Both partial (Lindeboom et al. 2001) and Nissen Fundoplication (Staathof et al. 2001) reduces the occurrences of TLESRs. Thus combining both the procedures, SIPS with LF, can be a feasible option for the treatment of GERD in morbidly obese patients.

The advantage of this novel technique as the treatment for both obesity and GERD is that the addition of malabsorption with the sleeve and the intestinal bypass make its weight loss more reliable than a sleeve alone, and this compensates for the larger volumes involved in making the LF.

This technique has not been previously discussed. All patients who underwent this combined procedure absolutely did not want a gastric bypass because of its side effects and two were on chronic NSAID therapy. This approach, SIPS with LF, allowed the patients to preserve the efficacy of the LF while maintaining the greater weight loss seen with SIPS (Hess et al. 2005; Buchwald et al. 2004).

The GERD-HRQL used here, has the advantage over standard health status instruments for GERD including simplicity for patients, high compliance rates, and the ease of understanding by physicians (Velanovich et al. 1996). Among the observed results of the GERD-HRQL questionnaire, questions 1–6 were related to heartburn, a typical symptom that affects patient’s quality of life. In this study, all our patients had a heartburn score of less than 12 with mean score of 3.2, which exhibits the elimination of their symptoms. Items 7–9 referred to symptoms of dysphagia, odynophagia, and meteorism considered side effects of the procedure. In this study these items were the main reason in raising the final score. However these symptoms are well tolerated by the patients and show improvement over time after the operation (Balci and Turkcapar 2007; Hamdy et al. 2008). Item 10 was about the use of medication, and all of our patient reported 0 (no symptoms) to this question, revealing low or no need of medication and low impact on their quality of life. The last question of GERD-HRQL assessed the patient’s perception about their current health status into 3 levels of satisfaction. It was noted that all of our patients marked satisfied and were between 6 and 12 months after the operation. It’s also worth mentioning that our mean total score was 6.2, with more than half of the patients having a total score below 5. Lower GERD-HRQL total scores indicated good quality of life, suggesting that surgical treatment was effective. This modality is better than drug therapy when considering quality of life and patient satisfaction.

There are some limitations in this study. First is the small case number. This study is not meant to provide definitive superiority to LF or LRYGBP alone in the setting of obesity but as a possibility in patients who both LF and LRYGBP are not options for various reasons. Consequently predicting its widespread applicability to all bariatric patients with reflux is premature and awaits larger trials. Secondly, we could not evaluate endoscopy or pH testing post-operatively in our patients, which is fundamental to evaluate the effect of anti-reflux surgery. Though we could get GERD-HRQL questionnaires for all our patients, we could not compare the data pre and post-surgery. However limited, the data does point to good quality of life following this surgery and this small study should be a stimulus to others in different centers to offer this option who have not considered it before.

According to our short-term follow up after SIPS with LF, we clearly demonstrate that it has effective early weight loss and a satisfactory anti-reflux effect.

Some may call this combined procedure experimental. However, our hospital surgical committee felt that since we were combining two known procedures, no IRB was needed. Additionally, the Blue Cross definition of experimental is any procedure where the outcome is unknown and in this case the two procedures outcomes are well known. The fact that we combined them doesn’t alter the effect of either one as we demonstrated.

## Conclusion

In summary, our early results are encouraging. SIPS with LF, is a novel and technically feasible procedure that combines fundoplication and bariatric surgery that can have good anti-reflux effects and substantial weight loss during the short term follow up. Additional long term follow ups and larger study populations would be required to further evaluate the outcomes of this novel technique to see if it is applicable to all bariatric GERD patients or should be reserved for special circumstances like we presented in this paper.
